# Prevalence of Anemia Among Gynecologic Cancer Patients Who Received Chemotherapy, Radiotherapy, or a Combination of Both at King Abdulaziz Medical City, Jeddah

**DOI:** 10.7759/cureus.17613

**Published:** 2021-08-31

**Authors:** Atheer H Alghamdi, Rahaf I Niyaz, Hatim Al-Jifree, Muhammad A Khan, Lamyaa Alsalmi

**Affiliations:** 1 College of Medicine, King Saud bin Abdulaziz University for Health Sciences, King Abdullah International Medical Research Center, Jeddah, SAU; 2 Department of Oncology, King Abdulaziz Medical City, Ministry of the National Guard Health Affairs, Jeddah, SAU; 3 College of Medicine, King Saud Bin Abdulaziz University for Health Sciences, King Abdullah International Medical Research Center, Jeddah, SAU

**Keywords:** key words: anemia, chemotherapy, radiotherapy, gynecologic cancers, prevalence

## Abstract

Objective: The aim of this study is to calculate hemoglobin (Hb) levels and find the prevalence of anemia in gynecological cancer patients undergoing cancer treatments including chemotherapy and radiotherapy attending Princess Noorah Oncology Center in King Abdul Aziz Medical City in Jeddah.

Method: A cross-sectional chart review study was conducted in gynecological cancer patients receiving chemotherapy/radiotherapy to find the prevalence of anemia in the period between 2016 and 2018. All data were collected from the electronic medical records using a data collection sheet.

Results: A total of 107 female patients who had gynecologic cancers and received chemotherapy/radiotherapy were included. Ninety percent (90.7%) of them developed anemia during the treatment course.

Conclusion: The prevalence of anemia in patients with gynecological cancers during their active treatment was high. This is attributed to the chemotherapy and radiotherapy they were receiving that affected their Hb levels. Better monitoring and, in severe cases, blood transfusion could be beneficial.

## Introduction

Cancer has a life-threatening impact on populations across the world occupying the first four places in the ranking of leading causes of death in different countries worldwide [1]. According to the International Agency for Research on Cancer (IARC), the estimated global incidence of cancer in 2008 was 12.7 million new affected patients and the mortality was 7.6 million deaths [2]. In 2012, IARC predictions were 14.1 million new cases and 8.2 million cancer deaths [3], and these estimated numbers increased to reach 18.1 million and 9.6 million for incidence and deaths in 2018, respectively [1]. The agency is expecting these numbers to increase to reach 23.6 million new cases in 2030 [3]. This increase in cancer incidence over years is considered as a consequence of population growth, increased average age, and other factors like socioeconomic status. Cancer types that are related to the developmental status of the country are being shifted to other types that have no relation to poverty and occur in the developed countries instead, which is due to “westernization of lifestyle” [1].

Talking specifically about females, IARC reported that cervical, endometrial, and ovarian tumors are the most occurring gynecologic cancers with mortality rates of 3.3%, 0.9%, and 1.9% in 2018, respectively [1]. According to the stage, grade, and clinical aspects of cancer, the treatment plan will differ, and with these variations, therapy-associated complications may occur, one of which is chemotherapy-induced anemia that, based on studies outside Saudi Arabia, may arise in over 80% of patients [4], and according to World Health Organization (WHO) standards, females with hemoglobin (Hb) level below 12 g/dL and males below 13 g/dL (normal range: 13-16 g/dL in females and 14-17 g/dL in males) are considered anemic [5].

Anemia is a negative confounder in cancer patients receiving chemotherapy since the basis of chemotherapy and the goal of cancer treatment is to target fast-growing neoplastic cells. However, because these drugs used in chemotherapy do not have the ability to distinguish good from evil nor alliance from the enemy, it might attack other fast-growing cells in the body like hair follicles and the red blood cells causing the hemoglobin level to drop, leading to this confounder, anemia [6]. Adding to what was mentioned, other treatments like radiotherapy might be affected by this negative confounder too. The main goal of radiation therapy is to cause deoxyribonucleic acid (DNA) damage so that the cancer cells cannot proliferate and, therefore, die. One way to do so is the “Indirect Action,” which takes advantage of the free radicals made by the interaction between radiation and oxygen molecules to cause the DNA to be damaged [7]. When the patient is anemic, tumor susceptibility to this regimen and survival will be less, and the patient would become more likely to undergo reoccurrence [8].

Previous studies, one was done in 2002, and another one was done in the period between 2010 and 2013, showed convergent numbers in terms of chemotherapy-induced anemia incidences, in ovarian cancer patients in particular, and this is considered significant since both studies results exceeded 90% [4,9]. With the high incidence rates obtained previously, and the lack of such studies in our region, and for better monitoring of our patients, we took the lead in this matter and conducted this study which was concerned with measuring the prevalence of anemia among gynecological cancer patients during their active treatment of chemotherapy/radiotherapy in Princess Norah Oncology Center in King Abdulaziz Medical City in Jeddah. This study was aiming to fill the gap we had in order for a better understanding of the problem and for improvement of the healthcare in our hospital.

## Materials and methods

We conducted a cross-sectional retrospective chart review by collecting data using a data collection sheet that included demographic information of the patients and clinical information of cancer. This study was conducted in KAMC tertiary care center, Princess Noorah Bin Abdulrahman Oncology Center (PNOC) in Jeddah and was approved by the IRB.

Females with gynecological cancer (ovarian cancers, endometrial cancers, cervical cancers, and gestational trophoblastic disease) who completed their chemotherapy, radiotherapy, or a combination of both at KAMC over the last two years were included in this study to measure the prevalence of anemia, with the exclusion of female patients who did not receive chemotherapy or radiotherapy, and those who had surgical procedures only.

Non-probability convenience sampling technique was used, and our sample size was calculated by Raosoft sample size calculator to be 139 patients after considering a 5% margin of error, a confidence level of 95%, response distribution of 89.5%, and a population of 3588. Clinical information that included type, stage, histopathology of cancer, lymph nodes involvement, number of chemotherapy cycles, mean hemoglobin level of each patient, and whether patients were receiving radiotherapy or concurrent radiation therapy was collected through “BESTCare” health information system by three medical students from KSAU-HS, and then data was managed by using Excel for data entry and SPSS (IBM SPSS Statistics, SPSS, Inc., Chicago, Illinois) for data analysis. Quantitative statistics were presented by mean and standard deviation, considering a significance level of 0.05.

Statistical analysis

The information of patients that we collected in this study is nationality, age at diagnosis, presence of smoking history or passive smoking, and patient status after the treatment. Moreover, characteristics of the tumor were included such as tumor type, lymph node involvement, cancer stage, and grade. The characteristics and demographics of patients and their treatment were described and presented as percentages for categorical variables. In addition, continuous variables were reported by the mean and standard deviation (SD), considering a significance level of 0.05. All analyses were conducted using SPSS version 10.0 (IBM SPSS Statistics, SPSS, Inc., Chicago, Illinois).

## Results

Our target sample size was 139 patients from the years 2017 and 2018, however, we faced a shortage of data due to many reasons. To overcome this problem, we extended the period of our data to include one more year, so 2016 was added. Despite the lack of data we faced, we managed to collect 107 patients.

The mean age of the targeted patients was 52.5 years (±12.57 SD). Since the patients were attending a national hospital in Saudi Arabia, most of them were Saudis except for two. Moving to talk about smoking, 82 patients (76.6%) were non-smokers, though a lot of smoking information was missing (24 patients, 22.4%). As for passive smoking, we could not find any recorded data in any of the patients’ notes (Table 1).

**Table 1:** Profile of study participants. The main demographics of the sample; N=107, their mean age is 52.5 years with a standard deviation of 12.57, the eldest is 80 years old and the youngest is 15 years old. Most of the samples are Saudis. The smoking history is not convenient with only one smoker and 24 to be unknown.

Demographic variables	Frequency N=107	Percentage
Mean ± SD for age	52 ± 12.57	
Nationality		
Saudi	105	98.1%
Non-Saudi	2	1.9%
History of smoking		
Smoker	1	0.9%
Non-smoker	82	76.6%
Unknown	24	22.4%

We collected Hb levels before starting any cancer treatment to the patients with a mean level of 11.7 g/dL ± 1.59 SD, and as defined by the WHO, Hb levels under 12 g/dL were considered anemic. Based on the previous definition, we noticed that 55.7% of patients (59 patients) were already anemic before starting their cancer treatment, while a percentage of 44.3 (n=47) were not. When talking about the mode of therapy, out of our 107 patients, 105 (98.1%) received chemotherapy, and only 29 patients (27.1%) took radiotherapy. Two patients (1.9%) did not receive chemotherapy, and most patients (78 patients, 72.9%) did not take radiotherapy. As for radiotherapy status, 25 completed their sessions (86.2%) and 4 did not (13.8%).

After each cycle of chemotherapy, Hb levels were collected and its mean appears to be 10.5 g/dL ± 1.2 SD. By looking at the McNemar test table (Table 2), it shows that out of the 47 patients who were having normal Hb levels before the initiation of their treatment, 39 patients (83.0%) developed anemia during the course of therapy, and the remaining eight patients (17.0%) continued to be normal. Only two persons (3.4%) of 59 people who were anemic ever before the start of the treatment got their Hb levels back to normal after treatment, while the other 57 patients (96.6%) remained anemic. Reaching a conclusion, the prevalence of anemia among gynecologic cancer patients who received chemotherapy alone or in combination with radiotherapy in our hospital is 90.7%.

**Table 2:** McNemar test table.

		Hb during treatment				p-value
		Anemia		Normal		
		n	%	n	%	
Hb before treatment	Anemia	57	96.6%	2	3.4%	<0.001
	Normal	39	83.0%	8	17.0%	
	Total	96	90.6%	10	9.4%	

Out of the most common histopathological types of gynecologic cancers, adenocarcinoma was the most prevalent among our subjects included in this study with a percentage of 33.6%, which constitutes 36 persons of the sample. Other various types, like small cell carcinoma and granulosa, were to be the second most common types (28%, 30 persons), and 24.3% (26 persons) of our sample were of unknown type, followed by squamous cancers that are found to be the least common one (14%, 15 persons).

Among 107 female patients, ovarian cancer (43%, 46 persons) was the most common classification noticed, followed by endometrial cancer, cervical cancer, other types of cancer, and uterine cancer with percentages of 29, 20.6, 5.6, and 1.9, respectively (Table 3). Thirty (28%) of our cancer patients were in stage 3C of cancer when they started the treatment, and stages 1C and 2C are the least commons comprising a percentage of 0.9% of each. Moving to cancer grade, the majority of the patients have unknown cancer grades (58.9%). High-grade cancers such as IVA and IVB are more prevalent with a total percentage of 18.7% (Table 4).

**Table 3:** Tumor type of the sample. Out of N=107, ovarian cancer is the most frequent, and uterine cancer appears to be the least frequent type.

Tumor type (n=107)	n	%
Ovarian cancer	46	43.0
Endometrial cancer	31	29.0
Cervical cancer	22	20.6
Uterine cancer	2	1.9
Other	6	5.6

**Table 4:** Stage and grade of study sample tumors. Stage 3C, which is considered a high tumor stage, occupied the first place in repetition, and stages 1C and 2C were at last place equally. As for the grade, lots of data were missing (unknown), however, IB-2 grade happened to be the least presented.

Stage (n=107)	n	%	Grade (n=107)	n	%
1A	7	6.5	IA	2	1.9
1B	10	9.3	IB-1	1	0.9
1C	1	0.9	IB-2	3	2.8
2A	4	3.7	IIA	6	5.6
2B	11	10.3	IIIA	10	9.3
2C	1	0.9	IIIB	2	1.9
3A	4	3.7	IVA	11	10.3
3B	10	9.3	IVB	9	8.4
3C	30	28.0	Unknown	63	58.9
4A	3	2.8			
Unknown	26	24.3			

Lymph node involvement was seen in 30.8% of all subjects by either imaging (22.4%), surgical biopsies (5.6%), or clinical evidence (0.9%), and it was absent in 20.6% of them (Table 5). Regarding surgical tumor removal, 50 patients (46.7%) had bilateral salpingo-oophorectomy (bilateral removal of fallopian tubes and ovaries), and 52 (48.6%) patients had total abdominal hysterectomy TAH (complete removal of uterus and cervix). Omentectomy was done in 17.9% of the patients (Table 5).

**Table 5:** Relations between study variables. The table shows the relations between different variables and their statistical significances, whether they are statistically significant or not.

	Hb during treatment				p-value
	Anemia		Normal		
	n	%	n	%	
Tumor type					
Ovarian cancer	39	84.8%	7	15.2%	0.543
Endometrial cancer	29	93.5%	2	6.5%	
Cervical cancer	21	95.5%	1	4.5%	
Uterine cancer	2	100.0%	0	0.0%	
Other	6	100.0%	0	0.0%	
Histopathology type					
Squamous	14	93.3%	1	6.7%	0.935
Adenocarcinoma	32	88.9%	4	11.1%	
Other	28	93.3%	2	6.7%	
Unknown	23	88.5%	3	11.5%	
Lymph node involvement					
No	19	86.4%	3	13.6%	0.014
Clinical	1	100.0%	0	0.0%	
Surgical	5	83.3%	1	16.7%	
Imaging	23	95.8%	1	4.2%	
Lymphovascular space	0	0.0%	2	100.0%	
Unknown	49	94.2%	3	5.8%	
Bowel resection					
Yes	2	66.7%	1	33.3%	0.257
No	95	91.3%	9	8.7%	
BSO					
Yes	44	88.0%	6	12.0%	0.291
No	53	93.0%	4	7.0%	
Omentectomy					
Yes	16	84.2%	3	15.8%	0.255
No	80	92.0%	7	8.0%	
TAH					
Yes	46	88.5%	6	11.5%	0.336
No	51	92.7%	4	7.3%	
Other					
Yes	16	94.1%	1	5.9%	0.505
No	81	90.0%	9	10.0%	
Cancer stage					
1A	7	100.0%	0	0.0%	0.490
1B	9	90.0%	1	10.0%	
1C	1	100.0%	0	0.0%	
2A	4	100.0%	0	0.0%	
2B	10	90.9%	1	9.1%	
2C	1	100.0%	0	0.0%	
3A	4	100.0%	0	0.0%	
3B	10	100.0%	0	0.0%	
3C	23	76.7%	7	23.3%	
4A	3	100.0%	0	0.0%	
Unknown	25	96.2%	1	3.8%	
Cancer grade					
IA	2	100.0%	0	0.0%	0.939
IB-1	1	100.0%	0	0.0%	
IB-2	3	100.0%	0	0.0%	
IIA	6	100.0%	0	0.0%	
IIIA	9	90.0%	1	10.0%	
IIIB	2	100.0%	0	0.0%	
IVA	11	100.0%	0	0.0%	
IVB	8	88.9%	1	11.1%	
Unknown	55	87.3%	8	12.7%	

According to the last documentation in 2019 on BestCare files concerning patients’ statuses, 23 patients (21.5%) are alive and free of cancer. Forty-two (39.3%) patients passed away or had not been cured yet, while we lost contact with the rest (39.3%).

## Discussion

This study aimed to measure the prevalence of anemia among gynecological cancer patients during their active treatment of chemotherapy that was associated or not with radiotherapy in PNOC, in KAMC, Jeddah. This study was aiming to fill the gap we had to have a better understanding of the problem and for further improvement of the healthcare provided to our patients in our hospital. This study investigated the impact of cancer treatment on patients' Hb levels. We compared the mean hemoglobin levels of our patients with the World Health Organization (WHO) standard hemoglobin level to determine if our patients were anemic or not. This study found that the majority of our patients who received cancer therapy developed anemia during their treatment. The results indicate that anemia is a common hematologic complication of chemotherapy and radiotherapy treatments with high prevalence.

We studied the relationship between our variables (tumor type, histopathology type, lymph node involvement, types of surgeries, cancer stage, and grade) and our main confounder, anemia, and we found that all findings were statistically insignificant except the relation between the lymph node involvement and anemia (Table 5). This could be due to the advanced disease status, since the presence of lymph node invasion may suggest a metastatic disease, which is a relatively poor prognostic factor, and this is perhaps being correlated with more treatment cycles and methods. Since chemotherapy and radiotherapy are associated with higher levels of anemia among cancer patients, and a metastatic disease might require more regimens of these, it makes sense that LN involvement was related to higher levels of anemia.

A study by the European Cancer Anemia Survey (ECAS) was done in 24 European countries to determine the rate of anemia (defined as Hb <12 g/dL) concluded that 75% of patients treated with chemotherapy developed anemia [10]. In another randomized clinical trial study where they used a large oncology electronic medical record database, the anemia rate (defined as Hb <11 g/dL) was 20.9% at baseline and increased to 59.0% after chemotherapy regimens [11]. One more study with a total sample size of 4426 patients reported that 89.5% of patients with solid tumors developed moderate to severe anemia (defined as Hb <10 g/dL) during the course of chemotherapy. In this study, they found that among ovarian cancer patients, in particular, 98.4% developed anemia during their chemotherapy regimen [4]. In our study, we found similar results to previous studies; anemia is a common complication in cancer patients undergoing cancer therapies. Moreover, such a study has never been done in our medical city in Jeddah nor our region.

The prevalence we reached at the end of our study was reasonable since it matches the mechanism of cancer therapies and the human body's response to it. Hence, the results are considered complementary. Still, our hospital is trying its best to reduce the incidence and severity of this confounder and to find better ways in managing patients.

Limitations

Some of the problems we faced while collecting our data were the lack of patients’ information inserted in the BestCare system, lack of radiotherapy data including Hb values (which is the core value of our research), and the presence of patients who received their treatment outside our hospital. Nevertheless, the oncology department lost contact with some patients after they got diagnosed. We also needed to exclude some patients who did not meet our criteria. For example, we had to exclude patients who underwent solely surgical treatment of tumors and did not need any adjuvant nor neoadjuvant chemotherapy or radiotherapy, excluding elderly and end-stage patients who were not applicable for any treatment except for analgesics (Palliative care).

## Conclusions

In this study, we measured the effect of chemotherapy and radiotherapy on the Hb level of cancer patients and calculated the mean Hb level during the treatment regimen. Our results concluded that 90.7% of the sample size developed anemia during the treatment. The risk of developing anemia is higher in patients with lymph node invasion.

For future research, we suggest measuring the effect of different chemotherapy drugs and the number of cycles on the Hb level. Nevertheless, knowing the degree and grade of anemia is important in the management. Adding to what has been mentioned, and looking at the targeted sample size in this study, new research can be conducted in the future to involve a bigger sample size and wider categories of patients to better represent the population.
